# Book Reviews

**Published:** 1896-06

**Authors:** 


### BOOK REVIEWS.

---

TWENTIETH CENTURY PRACTICE. AN INTERNATIONAL ENCYCLOPEDIA OF MODERN MEDICAL SCIENCE. By Leading Authorities of Europe and America. Edited by Thos. L. Stedman, M.D., New York City. Vol. V. DISEASES OF THE SKIN. New York, Wm. Wood & Co., 1896.

This is a large volume, the subject-matter of which is handled by men selected from the dermatological field for their fitness for the special work assigned them.

After the anatomy of the skin, the different diseases are taken up in the following order of classification: Parasitic Diseases—Animal, Vegetable; Erythematous Affections; Eczema and Dermatitis; Squamous Affections; Papular Affections; Bullous Affections; Pustular Affections; Phlegmonous and Ulcerative Affections; Diseases of the Sebaceous Glands; Diseases of the Sweat Glands; Diseases of the Hair and Nails; Benign Neoplasms; Xeroderma Pigmentosum; Dermato-Neuroses.

The bibliography is quite full.

One case of Xerodema Pigmentosum should have been credited to M. B. *Hutchins* not M. B. *Hutchinson.*

Some parts of the text are very full. Other parts seem deficient, as the tuberculoses; Cancerous Diseases of the Skin, etc.

The book fills the place intended for it. M. B. H.

---

VOICE-BUILDING AND TONE-PLACING. By H. Holbrook Curtis, M.D., Fellow of the New York Academy of Medicine, etc. Price $2.00. D. Appleton & Co., 72 Fifth ave., New York.

In this book the theory of singing is thoroughly and capably handled. The anatomy and physiology of the larynx, respiration, and registers of the human voice are scientifically discussed, and many valuable hints on voice-building are given. A casual perusal of this work indicates that it will supply a long-felt need among singers, *i. e.*, the comprehension of the vocal art from a scientific standpoint. This work deals purely with the vocal mechanism and does not touch upon the psychology of singing, which is a point decidedly in its favor. W. O.

---

INFANTILE MORTALITY DURING CHILDBIRTH, AND ITS PREVENTION. By A. Brothers, M.D., Visiting Gynecologist to Beth Israel Hospital, New York, etc. Being the William T. Jenks Prize Essay of the College of Physicians of Philadelphia for 1895. Published by P. Blakiston, Son & Co., 1012 Walnut St., Philadelphia.

The object of this elaborate essay is to detail the causes contributing to the death of the child preceding, during, or immediately after childbirth, as well as to call attention to the means now at the physician's disposal for the diminution of infantile mortality. The large amount of clinical material in obstetrics and pediatrics to which the author has had access for ten years has peculiarly qualified him for this essay, and we are sure that it well deserved the prize of the Jenks Memorial Fund. It is well worth the careful reading and study of every practitioner. A full bibliography appears at the end of every chapter.

---

INTERNATIONAL MEDICAL ANNUAL. A Work of Reference for Medical Practitioners. Fourteenth year. 1896. Price $2.75. E. B. Treat, Publisher, 5 Cooper Union, New York.

Following the plan of its predecessors, the 1896 Medical Annual presents a good digest of important medical and surgical material for the preceding year. The volume is even larger and more complete than heretofore. It contains a very good article on the Roentgen photography, with some suggestions as to its uses in surgery. The book is carefully edited, and, like previous volumes, will be found very useful to the practitioner.

---

TRANSACTIONS OF AMERICAN DERMATOLOGICAL ASSOCIATION. NINETEENTH ANNUAL MEETING.

We have received of Dr. Chas. Warren Allen, Secretary, a copy of the above. It is excellently got up and arranged and contains some beautiful plates. The book is full of instructive reading. The Secretary will please accept our thanks for the copy sent us.

